# KIF20A as a driver of anti-PD-1 resistance via PD-L1 downregulation in NSCLC: a biomarker validation and tumor microenvironment analysis

**DOI:** 10.1186/s12885-025-15221-6

**Published:** 2025-11-20

**Authors:** Tianyu Su, Lin Li, Chaonan Jing, Lei Chen, Xiang Wang

**Affiliations:** 1 Department of Oncology, Xuzhou Central Hospital, Xuzhou, 221002 Jiangsu China; 2 Department of Oncology, Shanghai Medical College, Fudan University Shanghai Cancer Center, Fudan University, Shanghai, 200032 China; 3 Jiangsu Key Laboratory of New Drug Research and Clinical Pharmacy, Xuzhou Medical University, Xuzhou, 221004 Jiangsu China; 4 The Xuzhou Clinical School of Xuzhou Medical University, Xuzhou, 221002 Jiangsu China

**Keywords:** NSCLC, KIF20A, PD-L1, Immunotherapy resistance, Tumor microenvironment

## Abstract

**Background:**

Immune checkpoint inhibitors targeting PD-1 show limited efficacy in non-small cell lung cancer (NSCLC) due to primary resistance. KIF20A, a cell cycle regulator implicated in chemotherapy resistance, may influence tumor immunity, but its role in anti-PD-1 resistance remains unclear.

**Methods:**

We performed comprehensive bioinformatics analyses to identify KIF20A as a resistance-associated hub gene. Clinical validation was performed in 106 NSCLC patients receiving anti-PD-1 therapy. KIF20A protein expression was assessed by immunohistochemistry (IHC), and tumor microenvironment (TME) profiling was performed using multiplex immunofluorescence (mIHC). Statistical analyses included chi-square tests, Kaplan-Meier survival, Cox regression, and Spearman correlation.

**Results:**

KIF20A was significantly upregulated in NSCLC versus adjacent tissues (63.2% vs. 13.2%, *P* < 0.001) and associated with lymph node metastasis, poor differentiation, and advanced stage (*P* < 0.05). High KIF20A expression correlated with primary resistance to PD-1 blockade (*P* = 0.019) and shorter post-immunotherapy overall survival (HR = 3.40, *P* = 0.016). Crucially, KIF20A-high tumors exhibited reduced PD-L1⁺ tumor cell density (375.4/mm² vs. 864.8/mm² in KIF20A-low tumors, *P* = 0.0002), with an inverse correlation (*r*=-0.249, *P* = 0.01). Patients with combined KIF20A-high/PD-L1-low expression had the worst prognosis (HR = 6.61, *P* = 0.030).

**Conclusions:**

KIF20A drives primary anti-PD-1 resistance in NSCLC through PD-L1 suppression and independently predicts poor survival. The KIF20A/PD-L1 signature stratifies patient risk, positioning KIF20A as both a prognostic biomarker and a therapeutic target to overcome immunotherapy resistance.

**Graphical Abstract:**

**Figure:**
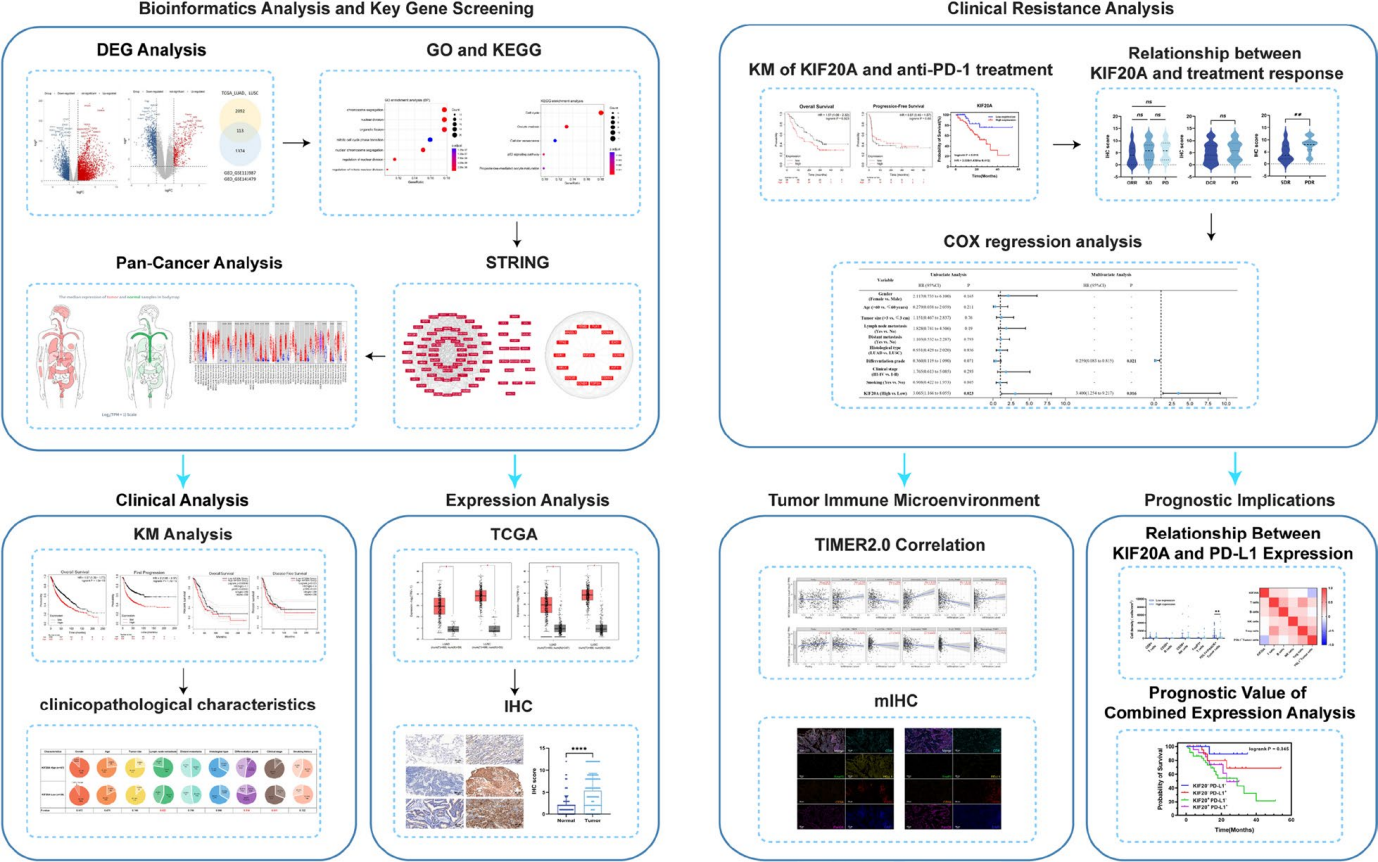
This study integrated bioinformatics analysis with clinical cohort validation to systematically explore the role of KIF20A in immunotherapy resistance in NSCLC. TCGA and GEO datasets were analyzed to identify resistance-associated genes. Clinical validation included IHC, multiplex immunofluorescence (mIHC), and survival analysis in 106 NSCLC patients treated with anti-PD-1 therapy.

**Supplementary Information:**

The online version contains supplementary material available at 10.1186/s12885-025-15221-6.

## Background

Lung cancer poses a significant worldwide health challenge and is the leading cause of cancer incidence and mortality globally. Accounting for 12.4% of all new cancer cases worldwide (over 2.5 million) and 18.7% of global cancer deaths (nearly 1.8 million) in 2022, lung cancer was a major contributor to the global cancer burden [1]. NSCLC constitutes >85% of all lung malignancies [2]. Conventional therapies—surgery, chemotherapy, radiotherapy, and targeted agents—face significant limitations: surgical resection is feasible in only 30% of early-stage patients [3], while platinum-based chemotherapy for advanced disease yields objective response rates (ORR) < 30% and a median overall survival of 8–10 months, often with severe hematologic toxicity [4]. Although targeted therapies against EGFR, ALK, and other driver genes improve outcomes in selected populations (15–20% of NSCLC cases), acquired resistance remains inevitable [5].

The advent of immune checkpoint inhibitors (ICIs), notably anti-PD-1 monoclonal antibodies, has fundamentally altered the therapeutic approach to NSCLC [6, 7]. PD-1 interacts with its ligand PD-L1tme, dampening T cell-mediated cytotoxicity [8]. Pivotal trials (e.g., KEYNOTE-024 [9], CheckMate 026 [10]) established anti-PD-1 agents ± chemotherapy as first-line therapy for PD-L1-high or driver-negative NSCLC, elevating 5-year survival to 23–31%. Nevertheless, primary or acquired resistance limits clinical benefit in most patients [11], underscoring the urgent need to elucidate resistance mechanisms and identify predictive biomarkers.

PD-1 resistance arises from tumor-intrinsic adaptations and immune microenvironment remodeling. Tumor cells evade immune recognition through antigen presentation defects (e.g., HLA-I loss) [12], oncogenic pathway activation (e.g., STK11/LKB1 mutations driving metabolic reprogramming) [13], and epigenetic silencing of tumor antigens [14]. The immunosuppressive tumor microenvironment, characterized by infiltrating regulatory T cells (Tregs), M2 macrophages [15], compensatory upregulation of LAG-3/TIM-3 [16], and cancer-associated fibroblast (CAF)-mediated barriers via CXCL12-dependent MDSC recruitment [17, 18], further compromises ICI efficacy. Notably, dysregulated cell cycle control contributes to resistance through dual mechanisms: genomic instability driven by p53 dysfunction or chromosomal aberrations depletes neoantigens and impairs T cell recognition [19–22], while mitotic kinases (e.g., Aurora A) recruit MDSCs and promote T cell exhaustion [23]. Despite these advances, the direct role of cell cycle regulators in anti-PD-1 resistance remains underexplored.

Kinesin Family Member 20 A (KIF20A), A motor protein bound to microtubules that is crucial for cytokinesis and cell progression [24, 25], is overexpressed in multiple malignancies and correlates with aggressive phenotypes, metastasis, and chemoresistance. KIF20A modulates therapeutic susceptibility by regulating mitotic catastrophe [26], ferroptosis [27], and apoptosis [28]. Its tumor-restricted expression and association with poor prognosis [29, 30] position KIF20A as a potential neoantigen [31, 32] and therapeutic target. Phase I/II trials of KIF20A-derived peptide vaccines show promise in pancreatic cancer [33–35], highlighting its immunogenic potential. Recent evidence implicates cell cycle genes in TME modulation [36], yet the interplay between KIF20A, anti-PD-1 resistance, and immune contexture in NSCLC remains unknown.

In this study, we integrated multi-omics bioinformatics analysis with clinical cohort validation to delineate the role of KIF20A in NSCLC. Our results demonstrate that KIF20A overexpression correlates significantly with adverse clinicopathological features and serves as an independent predictor of poor prognosis. Crucially, KIF20A upregulation confers primary resistance to anti-PD-1 therapy and exhibits an inverse association with PD-L1 expression. Mechanistic exploration reveals that KIF20A orchestrates an immunosuppressive tumor microenvironment characterized by diminished PD-L1^+^ tumor cell density. These findings establish KIF20A as a dual biomarker for prognostic risk stratification and immunotherapy response prediction. Furthermore, they unveil its therapeutic potential as a target to overcome immune checkpoint blockade resistance, providing a rationale for combinatory regimens integrating KIF20A inhibition with PD-1/PD-L1 axis modulation.

## Materials and methods

### Identification of anti-PD-1 resistance-associated genes

TCGA-derived RNA-seq datasets of NSCLC and matched adjacent tissues were analyzed for differentially expressed genes (DEGs) using the limma R package [37]. Anti-PD-1 treatment-related transcriptomes were obtained from GEO (GSE113987, GSE141479). Venn analysis identified overlapping DEGs between TCGA lung cancer and GEO immunotherapy cohorts.

### Functional enrichment and PPI network construction

GO and KEGG enrichment analysis of the overlapping DEGs was conducted using DAVID (https://davidbioinformatics.nih.gov/). Construction of protein-protein interaction (PPI) networks was carried out via STRING (https://cn.string-db.org/, confidence score > 0.7), followed by visualization in Cytoscape. Hub gene identification was performed using the CytoHubba plugin.

### Pan-cancer expression and immune infiltration analysis

TIMER2.0 (http://timer.cistrome.org/) [38]: Compared KIF20A expression across 33 cancer types (Gene_DE module) and evaluated correlations between KIF20A and immune cell infiltration (Immune Association module; partial correlation, *P* < 0.05). GEPIA2 (http://gepia2.cancer-pku.cn/) [39]: Validated KIF20A expression in NSCLC vs. normal lung (TCGA + GTEx; t-test, *P* < 0.05).

### Survival analysis

Prognostic significance was assessed via Kaplan-Meier Plotter (http://kmplot.com/analysis) [40]. NSCLC cohorts were stratified by median KIF20A expression. Overall survival (OS), progression-free survival (PFS), and post-progression survival (PPS) were analyzed (log-rank test; HR with 95% CI).

### Patient cohort and clinical specimens

Formalin-fixed paraffin-embedded (FFPE) tumor and paired adjacent tissues (>1 cm from tumor margin) were collected from 106 NSCLC patients undergoing radical resection at Xuzhou Central Hospital between January 2020 and January 2024. Inclusion criteria: Histopathologically confirmed NSCLC; No prior radiotherapy, chemotherapy, immunotherapy, or targeted therapy before surgery; Received immune checkpoint inhibitor-based systemic therapy postoperatively; Complete clinicopathological records and follow-up data. Clinicopathological Variables included sex, age, smoking history, differentiation grade, tumor size, TNM stage [41], lymph node/distant metastasis, and treatment response (RECIST v1.1) [42]. All patients received anti-PD-1 therapy with Tislelizumab and were evaluated for response to immunotherapy. OS was measured from the date of surgery to death or last follow-up. This study received approval from the Biomedical Research Ethics Committee of Xuzhou Central Hospital (Approval No: XZXY-LK-20240129-0024).

### Immunohistochemical staining

Following deparaffinization, 4-µm FFPE sections underwent antigen retrieval (EDTA, pH 9.0) and were subsequently incubated overnight at 4 °C with anti-KIF20A primary antibody (1:200; Proteintech, 67190-1-Ig). Detection was performed using a secondary antibody and DAB chromogen. Slides were counterstained with hematoxylin. Two blinded pathologists assessed positive cell percentage (0–4) and staining intensity (0–3). Immunoreactive score (IRS) = intensity × percentage. Scores of 0–1, 2–3, 4–6, and 8–12 were designated as “-“, “+”, “++”, and “+++”, respectively. Target protein expression levels were categorized as low (“-” or “+”) or high (“++” or “+++”). High expression was defined as IRS > 3 (Table S1).

### Multiplex immunofluorescence (mIHC)

Sequential mIHC staining was performed using the Krast 600 Automated Staining System (Krast Biotech) with Krast Multicolor Fluorescence Detection Kit to visualize the markers of PD-L1, B cells, Tregs, NK cells, CD8^+^ T cells and PD-L1^+^ tumor cells through staining the PD-L1, CD20, FoxP3, CD56, CD8, PanCK, DAPI. The primary antibodies and staining procedures are detailed in Supplementary Table S2. Refer to Supplemental Methods for additional details on processing and computer-assisted quantitative analyses.

### Statistical analysis

SPSS (v27.0), GraphPad Prism 9.5, and R (v4.5.1) were used for statistical analysis. The association between KIF20A expression and cancer development, patient clinicopathological characteristics, and treatment efficacy was analyzed using the chi-square test or Fisher’s exact test, as appropriate. Survival curves were constructed via the Kaplan-Meier method, with differences assessed using the log-rank test. Univariate and multivariate Cox regression analyses were conducted to identify independent prognostic factors in the cohort of 106 NSCLC patients. Spearman’s rank correlation was used to assess associations between KIF20A expression and tumor-infiltrating immune cell levels or PD-L1 expression. All statistical tests were two-sided, with statistical significance defined as *p* < 0.05.

## Results

### Identification of KIF20A as a core regulator in NSCLC immunotherapy resistance

**Fig. 1:**
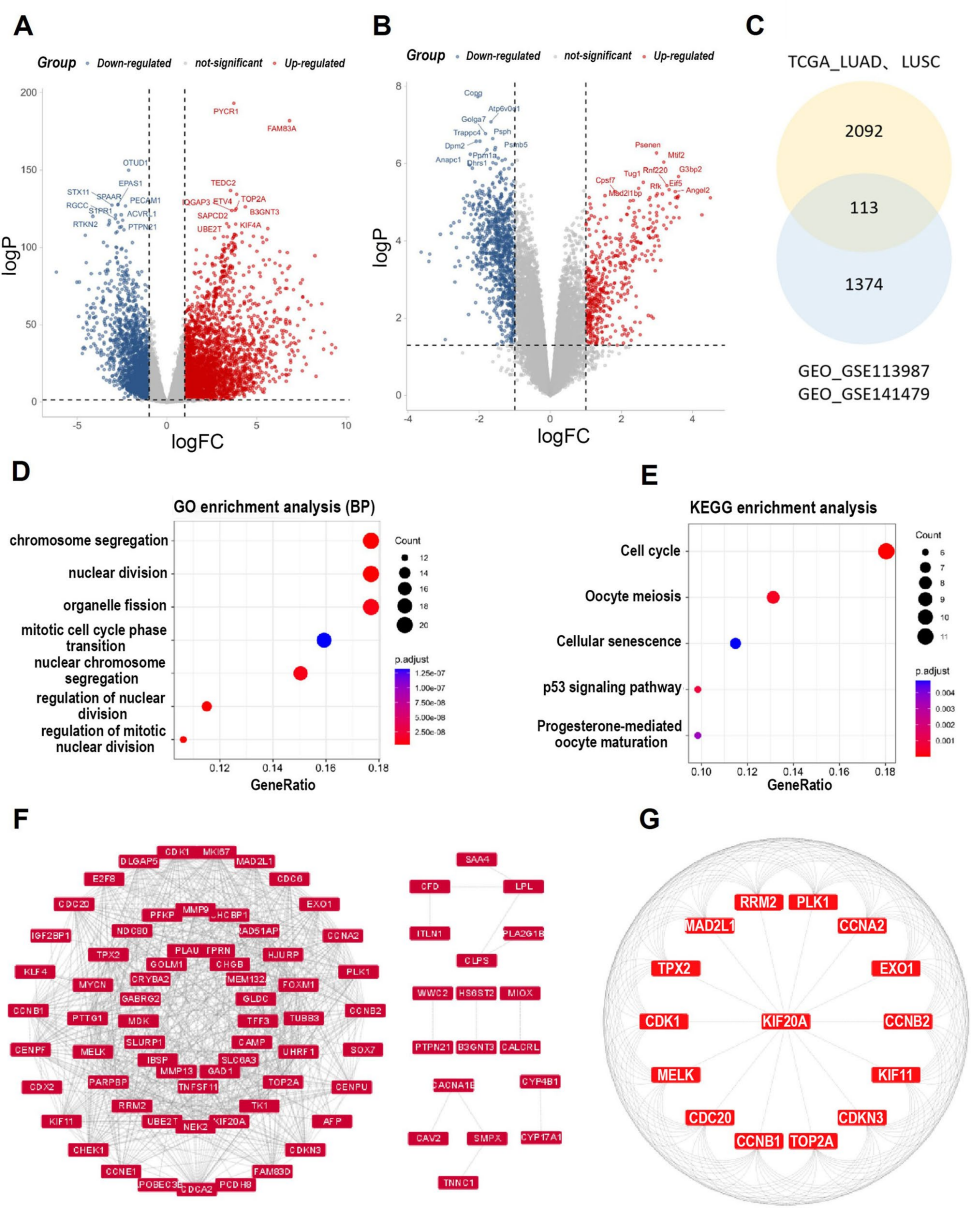
KIF20A is a hub gene linked to cell cycle regulation and anti-PD-1 resistance in NSCLC. **A** Volcano plot of DEGs in NSCLC vs. normal tissues (TCGA; *n* = 524 tumors, 58 normals; |log2FC|>1, *p* < 0.05). **B** DEGs in pre-anti-PD-1 vs. post-anti-PD-1 (GEO: GSE113987, GSE141479). **C** Venn diagram identifying 113 overlapping resistance-associated DEGs. **D** GO enrichment of overlapping genes. **E** KEGG pathway analysis. **F**, **G** PPI network (STRING) with KIF20A as a top hub gene

To explore molecules associated with immunotherapy resistance in lung cancer, mRNA sequencing data from lung cancer (LUAD, LUSC) and adjacent tissues were retrieved from the TCGA database, encompassing 524 tumor and 58 normal samples. Analysis revealed 2,205 differentially expressed genes (*p* < 0.05, |log2FC| >1) linked to lung cancer pathogenesis (Fig. 1A). Concurrently, transcriptomic data from NSCLC patients pre- and post-anti-PD-1 therapy were extracted from GEO datasets (GSE113987, GSE141479), identifying 1,487 DEGs (*p* < 0.05, |log2FC| >1) associated with PD-1 blockade response (Fig. 1B). Intersection analysis via Venn diagram identified 113 overlapping molecules implicated in anti-PD-1 therapeutic response in lung cancer (Fig. 1C).

GO enrichment analysis revealed significant enrichment of these 113 genes in chromosome segregation, nuclear division, and mitotic cell cycle transitions(Fig. 1D). KEGG pathway analysis further highlighted involvement in cell cycle, p53 signaling, and cellular senescence (Fig. 1E)—pathways critically associated with therapy resistance.PPI network construction (Fig. 1F) and CytoHubba analysis identified KIF20A as a top-ranked hub gene, interacting directly with core cell cycle regulators (CDK1, AURKB, PLK1) and mitotic effectors (TOP2A, FOXM1). This positioned KIF20A as a central coordinator of resistance-associated pathways.

### KIF20A exhibits pan-cancer upregulation and prognostic significance in NSCLC

**Fig. 2:**
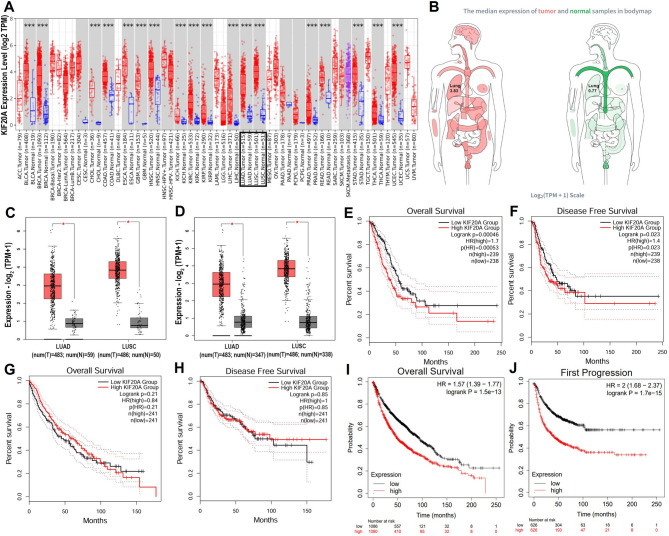
KIF20A is upregulated across cancers and predicts poor prognosis in NSCLC. **A** Pan-cancer KIF20A expression (TIMER2.0; red: tumor, black: adjacent normal tissues). **B** Pan-cancer KIF20A expression in bodymap (GEPIA2 -cohort). **C**, **D** The differential expression of KIF20A gene level between LUAD/LUSC and normal tissues in two validation cohorts (TCGA and TCGA + GTEx cohorts). **E**, **F** Kaplan-Meier survival in TCGA-LUAD and (**G**, **H**) LUSC with high- and low- gene expression of KIF20A. **I**, **J** Kaplan-Meier curves of survival in NSCLC patients (Kaplan-Meier Plotter- cohorts; *n* = 2166). *, *p* < 0.05; **, *p* < 0.01; ***, *p* < 0.001 and ns: not significant. HR: Hazard Ratio

After identifying KIF20A as the pivotal hub gene, we conducted an extensive pan-cancer analysis of KIF20A expression patterns across multiple tumor types using the TIMER2.0 platform (Fig. 2A), with a specific focus on NSCLC. KIF20A mRNA was significantly upregulated in multiple malignancies compared to matched normal tissues (*P* < 0.001).We further validated these findings using the GEPIA2 online platform. Consistent with the TIMER2.0 results, pan-cancer analysis confirmed that KIF20A expression was markedly higher in tumor tissues than in paired normal tissues across most cancer types, particularly in NSCLC. Notably, log₂-transformed KIF20A expression levels were approximately 4.97-fold higher in NSCLC tissues than in normal tissues (Fig. 2B; Fig. S1, S2). Subsequent analysis of TCGA data via GEPIA2 revealed significantly elevated KIF20A expression in both LUAD (tumor, *n* = 483 and normal, *n* = 59, *P* < 0.001; Fig. 2C) and LUSC (tumor, *n* = 486 and normal, *n* = 50, *P* < 0.001; Fig. 2C) compared to their respective normal counterparts. To enhance statistical power, we incorporated normal lung tissue data from the GTEx database (LUAD: *n* = 483 tumors vs. *n* = 347 normals; LUSC: *n* = 486 tumors vs. *n* = 338 normals). This expanded analysis confirmed that KIF20A expression remained significantly elevated in NSCLC tissues (*P* < 0.001, Fig. 2D). These results collectively indicate that KIF20A is closely associated with carcinogenesis and progression, with its overexpression suggesting a critical role in NSCLC pathogenesis.

We next evaluated the prognostic significance of KIF20A in NSCLC using clinical data from TCGA. Kaplan-Meier analysis indicated that elevated KIF20A expression significantly correlated with reduced OS and PFS in LUAD patients.(log-rank *P* < 0.05; Fig. 2E, F). However, this association was not observed in LUSC patients (Fig. 2G, H). To enhance statistical robustness, we analyzed a larger NSCLC cohort (*n* = 2,166) from the Kaplan-Meier Plotter database. Consistent with the LUAD-specific trend, high KIF20A expression correlated significantly with reduced OS in the combined NSCLC cohort (HR = 1.57, 95%, log-rank *P* = 1.5e-13; Fig. 2I). Similar negative associations were observed for time to first progression (FP; *n* = 1,252, HR = 2, log-rank *P* = 1.7e-15; Fig. 2J) and post-progression survival (PPS; *n* = 477, HR = 1.33, log-rank *P* = 0.0073; Fig. S3). Survival analysis revealed KIF20A as a robust prognostic biomarker.

### KIF20A overexpression in NSCLC: subcellular localization and clinical significance

**Fig. 3:**
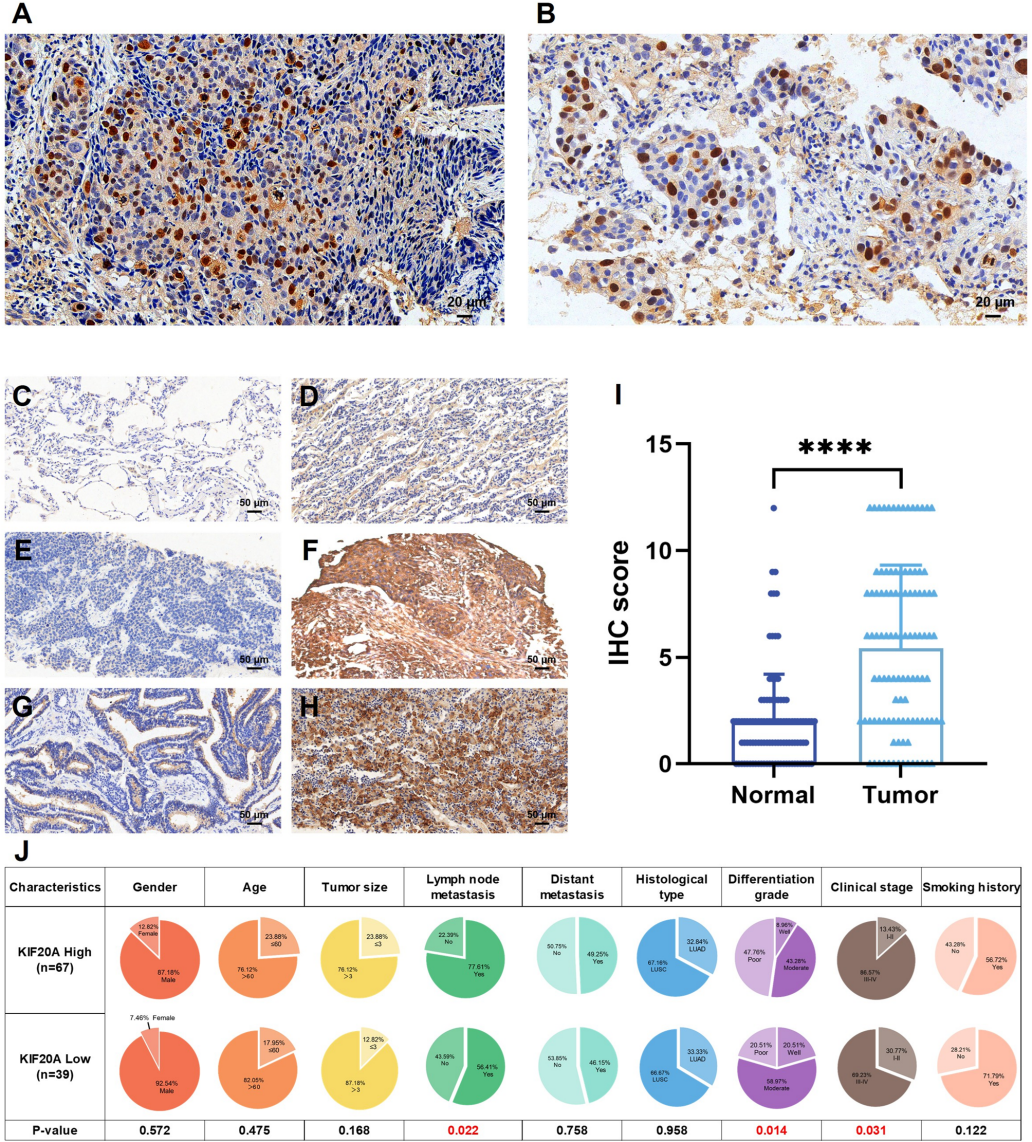
KIF20A protein overexpression correlates with aggressive clinicopathology in NSCLC. Positive expression of KIF20A in LUSC (**A**) and LUAD (**B**). Scale bars: 20 μm. Representative IHC: KIF20A is negative in paracancerous tissues (**C**, **D**), and is cytoplasmic/nuclear positive in LUSC (**E** weak/**F** strong) and LUAD (**G** weak/**H** strong). Scale bars: 50 μm. **I** KIF20A IHC scores (tumors vs. adjacent). **J** High KIF20A associates with lymph node metastasis, poor differentiation, and advanced stage (*p* < 0.05; chi-square test). *, *p* < 0.05; **, *p* < 0.01; ***, *p* < 0.001 and ns: not significant

To validate KIF20A protein expression, we performed immunohistochemistry (IHC) on pathological specimens from NSCLC patients. Immunohistochemical analysis revealed predominant cytoplasmic and nuclear localization of KIF20A in NSCLC tissues (Fig. 3A, B). Consistent with mRNA results, the KIF20A-positive rate in NSCLC tissues was significantly higher than in adjacent normal lung tissues (63.21% vs. 13.21%; Supplementary Table S3). IHC revealed minimal KIF20A expression in adjacent normal tissues, which appeared unstained or pale yellow with negligible cytoplasmic staining (Fig. 3C, D). In contrast, weak staining was observed in a minority of LUSC tissues (Fig. 3E), while strong staining was present in both the cytoplasm and nucleus of most LUSC samples (Fig. 3F). A similar pattern was seen in LUAD tissues, with weak staining in a minority of cases (Fig. 3G) and strong staining in the majority of samples (Fig. 3H). As shown in Fig. 3I, the IHC intensity score for KIF20A was significantly higher in NSCLC tissues than in adjacent tissues. Patients with high KIF20A expression showed increased propensity for lymph node metastasis, poor differentiation, and advanced-stage disease (Fig. 3J; Supplementary Table S4). Nevertheless, given the relatively modest sample size and inherent limitations of semi-quantitative analysis, multi-center studies with expanded cohorts and integration of additional potential factors are warranted. This comprehensive approach will enhance our ability to evaluate the predictive potential of KIF20A for NSCLC survival outcomes.

### Role of KIF20A in resistance to anti-PD-1 antibody therapy

**Fig. 4:**
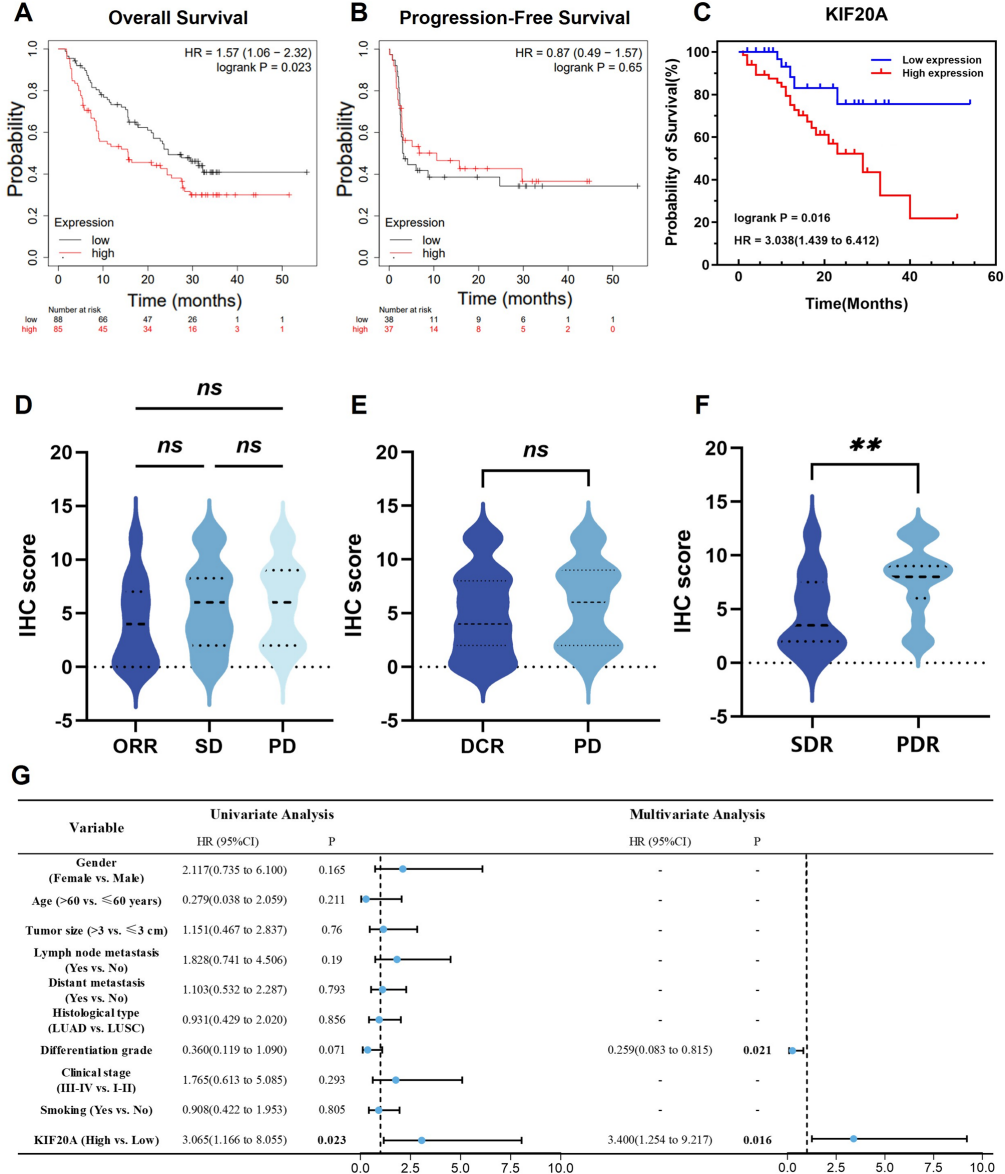
KIF20A drives primary resistance to anti-PD-1 therapy and predicts poor survival. **A**, **B** KM-Plotter: High KIF20A correlates with worse OS in anti-PD-1-treated solid tumors (nivolumab; HR = 1.57, *p* = 0.023) but not PFS (*p* = 0.65). **C** NSCLC cohort: High KIF20A confers shorter post-immunotherapy OS (HR = 3.038, *p* = 0.016). **D**, **E** The intensity of KIF20A showed no significant difference between the ORR and DCR groups. **F** Primary resistance cases show higher KIF20A vs. acquired resistance. **G** Multivariate Cox regression: KIF20A is an independent risk factor (HR = 3.400, *p* = 0.016). *, *p* < 0.05; **, *p* < 0.01; ***, *p* < 0.001 and ns: not significant. CR, Complete Response; PR, Partial Response; SD, Stable Disease; PD, Progressive Disease; ORR, Objective Response Rate; DCR, Disease Control Rate; PDR, Primary Drug Resistance; SDR, Secondary Drug Resistance; HR: Hazard Ratio

To determine KIF20A’s prognostic impact in anti-PD-1-treated solid tumors, we first conducted Kaplan-Meier survival analysis through the KM database. KIF20A expression showed a significant negative correlation with OS in patients receiving nivolumab (*P* = 0.023; HR = 1.57, 95% CI: 1.06–2.32; Fig. 4A). Conversely, KIF20A expression showed no significant association with PFS in this cohort (*P* = 0.65; Fig. 4B).

To further clarify its relationship with prognosis, we investigated the correlation between KIF20A expression and survival outcomes in NSCLC patients who received immunotherapy. Kaplan-Meier survival analysis demonstrated significantly reduced median OS in KIF20A-high NSCLC patients (27.5 months, 95% CI: 21.4–33.6) versus low-expression group(44.4 months, 95% CI: 36.8–51.9). This negative correlation was statistically significant, indicating that high KIF20A expression predicts poor prognosis following immunotherapy(*P* = 0.016; HR = 3.038, 95% CI: 1.439–6.412; Fig. 4C). Univariate Cox analysis confirmed KIF20A as an independent prognostic risk factor for OS (HR = 3.065, 95% CI: 1.166–8.055; *P* = 0.023). After adjusting for differentiation grade (selected by univariate *P* < 0.1), multivariate Cox regression validated KIF20A as an independent prognostic factor for OS (HR = 3.400, 95% CI: 1.254–9.217; *P* = 0.016; Fig. 4G).

We next evaluated the impact of KIF20A expression levels on treatment response in NSCLC patients. Among 106 NSCLC patients evaluated for anti-PD-1 antibody response, 25 achieved CR or PR (ORR = 23.6%), 38 had SD, and 43 had PD (DCR = 59.4%). KIF20A expression showed no significant correlation with ORR (*P* = 0.394; Supplementary Table S5) or DCR (*P* = 0.736; Supplementary Table S6). However, in the PD subgroup (*n* = 43), high KIF20A expression was strongly associated with primary resistance (84.2% vs. 15.8% in low expressors; *P* = 0.019; Supplementary Table S7). Immunohistochemical scoring further validated these findings: while KIF20A intensity did not differ significantly across ORR, SD, and PD groups (*P* > 0.05; Fig. 4D, E), primary resistance cases exhibited markedly higher KIF20A expression than acquired resistance cases (*P* < 0.05; Fig. 4F). This aligns with the observation that PD patients with primary resistance had a higher proportion of strongly KIF20A-positive tumors (Fig. S4).

### KIF20A expression modulates immune cell infiltration and spatial distribution in the NSCLC TME

**Fig. 5:**
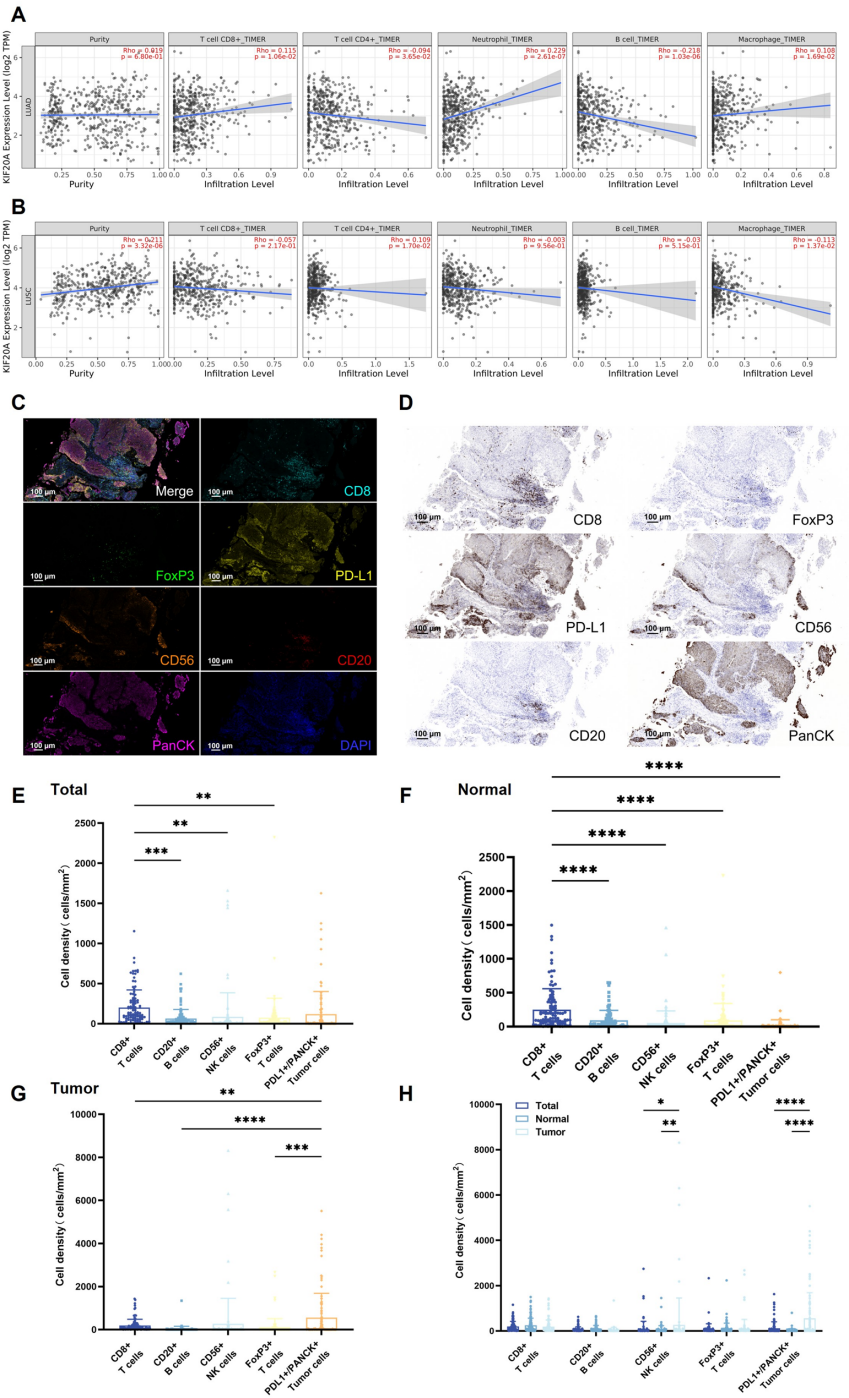
KIF20A expression shapes immune infiltration in NSCLC TME. **A**, **B** TIMER2.0: In LUAD, KIF20A positively correlates with CD8⁺ T cells, neutrophils, and macrophages but negatively with B cells and CD4⁺ T cells (partial correlation; *p* < 0.05). Opposite correlations in LUSC. **C**, **D** mIHC spatial mapping (representative images; scale bars: 100 μm). **E**-**H** Immune cell densities: Tumors show elevated PD-L1⁺ cells and NK cells (*p* = 0.0029) vs. adjacent tissue. CD8⁺ T cells dominate adjacent regions. *, *p* < 0.05; **, *p* < 0.01; ***, *p* < 0.001 and ns: not significant

To delineate the association between KIF20A expression and tumor immune infiltration, we performed TIMER2.0 analysis on TCGA-LUAD and LUSC cohorts. In LUAD, KIF20A expression exhibited a positive correlation with infiltration levels of CD8⁺ T cells (*r* = 0.115, *P* = 1.06e-02), neutrophils (*r* = 0.229, *P* = 2.61e-07), and macrophages (*r* = 0.108, *P* = 1.69e-02), while showing negative correlations with B cells (*r* = -0.218, *P* = 1.03e-06) and CD4⁺ T cells (*r* = -0.094, *P* = 3.65e-02) (Fig. 5A). Conversely, in LUSC, KIF20A expression positively correlated with CD4⁺ T cells (*r* = 0.109, *P* = 1.70e-02) but negatively correlated with macrophages (*r* = -0.113, *P* = 1.37e-02) (Fig. 5B). No significant associations were observed with neutrophils, CD8⁺ T cells, or B cells in LUSC (*P* > 0.05).

We next performed multiplex immunofluorescence (mIHC) on 106 paired NSCLC and adjacent normal tissues to map the spatial distribution of key immune subsets. mIHC results revealed a diverse array of immune cell types within the NSCLC tumor tissue, highlighting both the complexity of the tumor microenvironment and the potential roles of these immune cells in tumorigenesis and progression (Fig. 5C-E). Tumor-adjacent tissues were dominated by CD8⁺ T cells (density: 251.05/mm², *P* < 0.0001 vs. other subsets) (Fig. 5F). In contrast, tumor tissues exhibited a significant shift toward PD-L1⁺ tumor cells (555.44/mm² vs. 18.15/mm² in normal tissue; *P* < 0.0001) (Fig. 5G, H). Notably, NK cell infiltration was markedly elevated in tumors (267.63/mm² vs. 49.75/mm² in normal tissue; *P* = 0.0029).In contrast, no statistically significant difference was observed in the distribution patterns between CD20⁺ B cells and Foxp3⁺ Treg cells (*P* > 0.05; Fig. 5H).

### Inverse correlation between KIF20A and PD-L1 expression and combined prognostic value

**Fig. 6:**
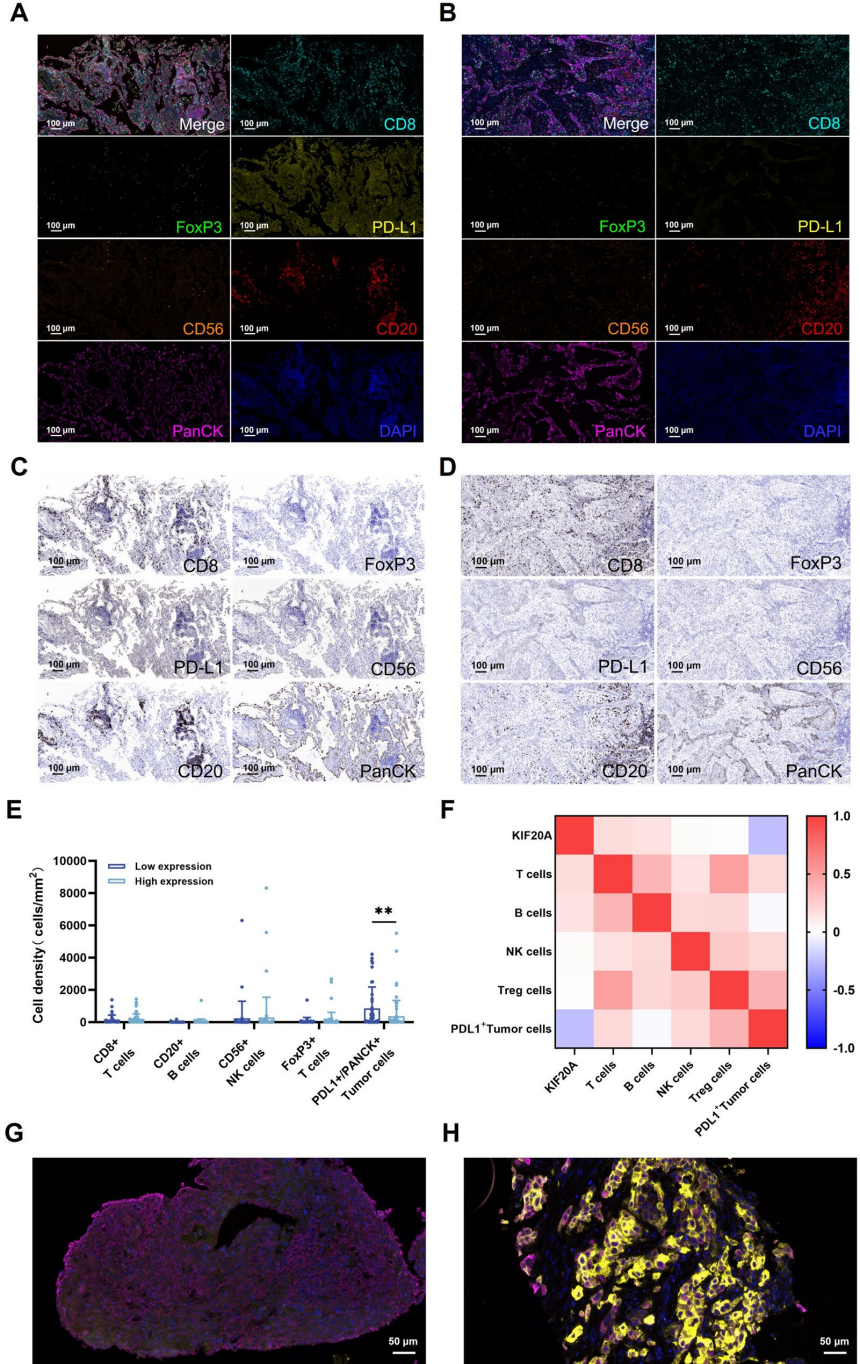
KIF20A inversely regulates PD-L1⁺ tumor cell density. mIHC of KIF20A-low (**A**, **C**) vs. KIF20A-high (**B**, **D**) tumors. Scale bars: 100 μm. **E** PD-L1⁺ tumor cell density is reduced in KIF20A-high tumors (*p* = 0.0002). **F** Spearman correlation confirms inverse KIF20A/PD-L1 relationship (*r*=-0.249, *p* = 0.01). **G**, **H** Representative images: Sparse PD-L1⁺ cells in KIF20A-high regions (**H**) vs. dense PD-L1⁺ clusters (yellow) in KIF20A-low areas (**G**). DAPI (blue), PanCK (Purple). Scale bars: 50 μm. *, *p* < 0.05; **, *p* < 0.01; ***, *p* < 0.001 and ns: not significant

To assess KIF20A-immunome associations, we stratified 106 NSCLC specimens into KIF20A-low and KIF20A-high cohorts based on IHC quantification (Fig. 6A-D). Multiplex immunofluorescence analysis demonstrated no significant differences in intergroup variation in infiltration densities of CD8⁺ T cells, NK cells, B cells, and Tregs (*P* > 0.05). Notably, however, PD-L1⁺ tumor cell density was significantly higher in KIF20A-low tumors compared to KIF20A-high tumors (864.8/mm² vs. 375.4/mm²; *P* = 0.0002; Fig. 6E). Spearman correlation analysis confirmed a significant inverse relationship between KIF20A and PD-L1 expression (Fig. 6F, Supplementary Table S8). Representative images demonstrated sparse PD-L1⁺ cells in KIF20A-high regions, contrasting with dense PD-L1⁺ clusters in KIF20A-low areas (Fig. 6G, H), suggesting potential regulatory crosstalk.

**Fig. 7:**
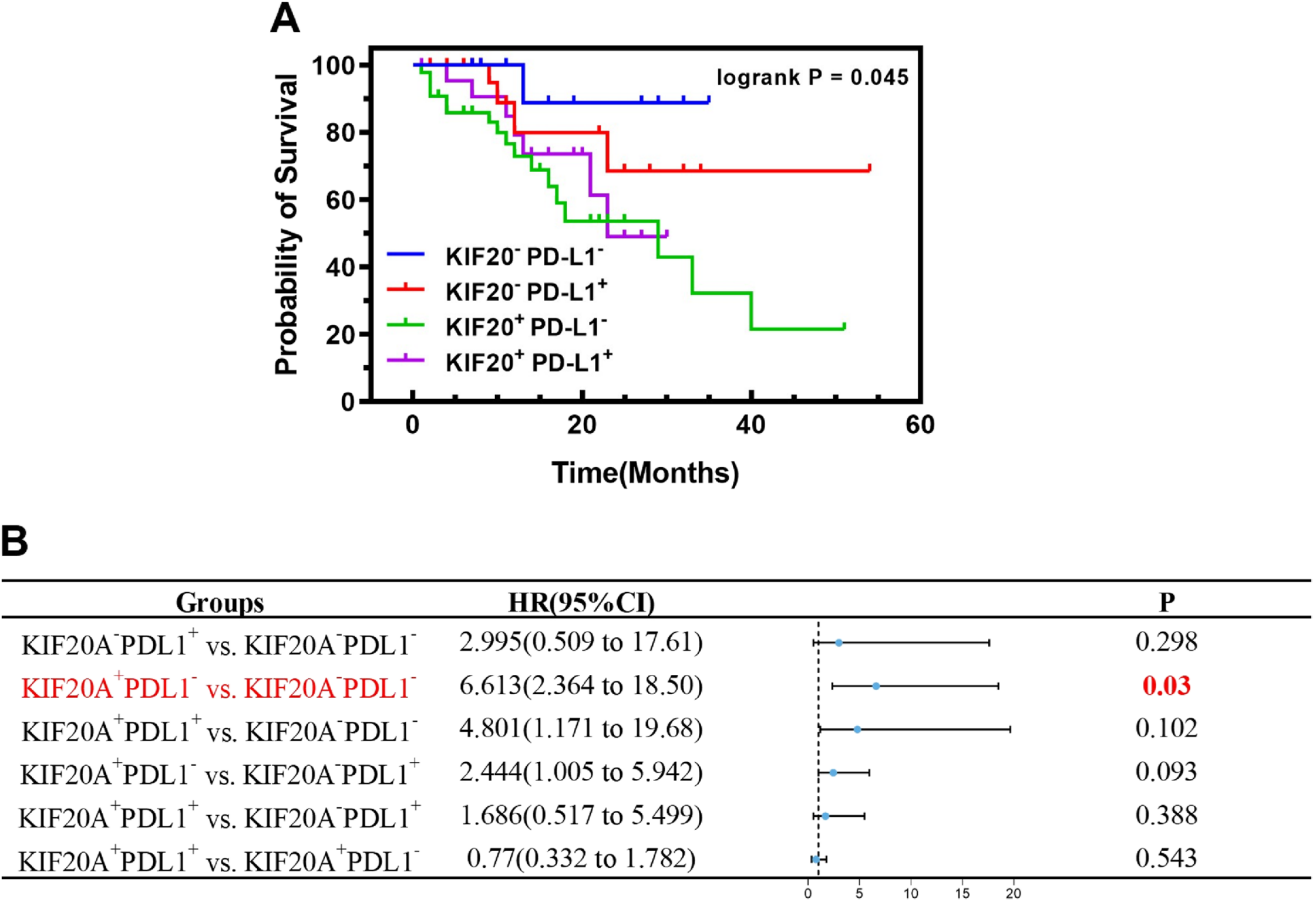
Combined KIF20A/PD-L1 signature stratifies prognosis in immunotherapy-treated NSCLC. **A** Kaplan-Meier analysis: KIF20A-high/PD-L1-low patients show worst OS (*p* = 0.045). **B** Cox regression analysis: KIF20A⁺/PD-L1⁻ group has highest mortality risk (HR = 6.61, *p* = 0.030 vs. KIF20A⁻/PD-L1⁻).*, *p* < 0.05; **, *p* < 0.01; ***, *p* < 0.001 and ns: not significant. HR: Hazard Ratio

Kaplan-Meier analysis revealed distinct survival outcomes based on combined KIF20A/PD-L1 status (*P* = 0.045; Fig. 7A). Patients with KIF20A-high/PD-L1-low expression exhibited the poorest survival, while the KIF20A-low/PD-L1-low subgroup had the most favorable prognosis. Pairwise comparisons demonstrated significantly elevated mortality risk in the KIF20A⁺/PD-L1⁻ group (HR = 6.613; 95% CI: 2.364–18.50; *P* = 0.030). The KIF20A⁺/PD-L1⁺ group showed a non-significant trend toward higher risk versus KIF20A⁻/PD-L1⁻ controls (HR = 4.801; 95% CI: 1.171–19.68; *P* = 0.102). No other intergroup differences reached statistical significance (Fig. 7B).

## Discussion

This study integrates bioinformatics analyses with clinical validation to systematically elucidate the role of KIF20A in NSCLC. We demonstrate that KIF20A is significantly overexpressed in NSCLC tissues and correlates with poor prognosis, primary resistance to anti-PD-1 therapy, and suppressed PD-L1 expression. The study identifies KIF20A as both a resistance biomarker and actionable target, offering translational strategies to augment anti-PD-1 efficacy. Below, we contextualize our key discoveries, explore their mechanistic implications, and discuss clinical relevance and limitations.

Kinesin Family Member 20 A (KIF20A), a member of the kinesin superfamily, plays a critical role in cell division by transporting chromosomes during mitosis [24]. Elevated KIF20A expression has been documented in multiple cancer types [43–45] and is closely associated with tumor cell proliferation, invasion [46], and drug resistance [26]. Our study systematically revealed a dual role for KIF20A in NSCLC through integrated analysis of TCGA and GEO databases, alongside clinical samples. First, KIF20A expression was significantly elevated in NSCLC tissues compared to normal controls. This high expression correlated strongly with poor histological differentiation, advanced clinical stage, and lymph node metastasis. Critically, survival analysis demonstrated that NSCLC patients receiving immunotherapy experienced significantly shorter overall survival in the KIF20A-high group. Subsequent multivariate Cox regression analysis confirmed KIF20A as an independent prognostic risk factor, with high expression conferring a 3.400-fold increased risk of mortality. This finding aligns with studies in breast [30] and pancreatic cancer [46], where KIF20A accelerates tumor progression by regulating mitotic processes, and its expression is inversely correlated with patient survival. Second, and more novel, our study reveals that KIF20A-high NSCLC patients exhibit significantly increased primary resistance to PD-1 monoclonal antibody therapy. This correlative finding suggests KIF20A may be associated with impaired immunotherapy response, potentially by modulating intrinsic tumor cell adaptive mechanisms. Functional enrichment analysis further indicated KIF20A involvement in cell cycle regulation and the p53 signaling pathway. This enrichment implies a potential and speculative mechanism whereby KIF20A could induces genomic instability, leading to neoantigen depletion and impaired T-cell recognition, thereby promoting therapeutic resistance. This finding offers a fresh perspective on PD-1 blockade resistance: while traditional research focuses on PD-L1 loss or T-cell exhaustion [47], our results highlight that dysregulation of cell cycle-related genes like KIF20A may may be associated with immune escape via pathways such as p53 signaling. However, the precise causal relationship remains to be established through direct experimental validation.

The tumor microenvironment, which critically influences ICI responsiveness, drives immune evasion and therapy resistance via multiple mechanisms: impaired immune checkpoint signaling (PD-1/PD-L1, CTLA-4/B7), defective T-cell infiltration, immunosuppressive cell expansion (MDSCs, TAMs), and immunoediting-induced T-cell exhaustion [48–53]. Targeting these pathways may overcome anti-PD-1 resistance. Our bioinformatic analyses revealed histology-specific associations between KIF20A and immune infiltration. In LUAD, KIF20A correlated positively with CD8^+^ T cells, macrophages, and neutrophils, whereas in LUSC, it correlated with CD4^+^ T cells. This divergence likely reflects distinct TME profiles: in LUAD’s immunosuppressive TME, KIF20A-linked neutrophil/macrophage infiltration suggests recruitment of MDSCs or M2 macrophages, while its association with CD8^+^ T cells may indicate an “immune desert” phenotype, marked by functionally exhausted T cells, consistent with TGF-β-mediated T-cell exclusion [17]. In LUSC, the immune microenvironment exhibits adaptive immune escape. KIF20A expression positively correlates with CD4^+^ T cell infiltration and may suppress anti-tumor immunity by activating Treg cells. However, its specific regulatory mechanisms require further validation using single-cell RNA sequencing or spatial transcriptomics technologies. It is important to note a key discrepancy between these transcriptomic correlations and our mIHC findings. The bulk mRNA analysis from TIMER2.0 reflects the average expression from a mixed cell population and may be influenced by overall tumor cellularity or expression in non-immune stromal cells. In contrast, mIHC provides precise spatial and protein-level quantification within the tissue architecture. Our mIHC analysis did not show significant differences in immune cell densities (CD8⁺ T cells, NK cells, B cells, Tregs) based on KIF20A protein expression levels. This divergence suggests that the correlations observed at the transcriptome level may not directly translate to alterations in the spatial abundance of these immune cells as measured by protein markers. Instead, our mIHC data indicate that KIF20A’s primary effect in the TME appears to be tumor cell-intrinsic, notably the significant reduction in PD-L1⁺ tumor cell density, rather than through direct modulation of immune cell recruitment or exclusion. Critically, high KIF20A expression significantly reduced PD-L1^+^ cell density, contrasting with Aurora Kinase A’s MDSC-driven resistance [23]. Given PD-L1’s regulation by DNA damage response (DDR) pathways [54], we hypothesize, based on correlative data, that KIF20A-driven mitotic dysregulation might potentially attenuate DDR signaling, indirectly suppressing PD-L1 transcription. This proposed axis remains speculative and necessitates direct experimental investigation in future studies.

To elucidate the clinical significance of KIF20A and PD-L1, this study evaluated their combined prognostic value. Kaplan-Meier analysis demonstrated significantly worse OS in patients with KIF20A-high/PD-L1-low tumors, implying that KIF20A-driven PD-L1 suppression may compromise anti-PD-1 efficacy by attenuating checkpoint activation. Furthermore, the KIF20A-high/PD-L1-high subgroup also demonstrated a significantly increased risk of death compared to the KIF20A-low/PD-L1-low group (HR = 4.801), albeit lower than the risk observed in the KIF20A-high/PD-L1-low group. We hypothesize that high PD-L1 expression may partially restore the efficacy of PD-1 inhibitors by facilitating immune checkpoint interactions between tumor cells and T cells, thereby mitigating the KIF20A-driven oncogenic effects. These findings challenge the conventional view of PD-L1 solely as a positive predictive biomarker for immunotherapy. Consequently, we propose a novel hypothesis: KIF20A may function as a “proliferation-immunity evasion switch” in tumor cells, where its expression level determines whether tumor progression is predominantly driven by accelerated proliferation or immune suppression.

However, this study has several limitations that must be emphasized. First and foremost, the single-center design and relatively modest sample size (*n* = 106) limit the generalizability of our findings across diverse ethnic and geographical populations. Yet, this inherent limitation also defines the unique value and immediate implication of our work: the ethnic homogeneity of our cohort provides a focused validation of KIF20A specifically within a Chinese patient population, which carries a substantial proportion of the global NSCLC burden. Therefore, while broader multi-ethnic studies are essential to define the global utility of KIF20A, our findings strongly position it as a promising, population-specific biomarker with high potential for optimizing risk stratification and guiding combination immunotherapy strategies for Chinese patients. Future research should prioritize large-scale, prospective validation in multi-center Chinese cohorts to confirm its clinical utility. Success in such validation would establish KIF20A as a powerful tool for patient stratification and combination therapy development tailored to the Chinese population, even as its global relevance is explored in parallel. Second, Our mechanistic exploration relied primarily on bioinformatic analyses and clinical correlations; rigorous in vitro and/or in vivo experiments are required to elucidate the precise molecular pathway(s) through which KIF20A regulates PD-L1 expression.Third, the analysis of the TIME focused solely on static cell densities. We did not assess the functional states of immune cells influenced by KIF20A, nor did we quantify the secretion levels of key immunosuppressive factors. Finally, the impact of dynamic changes in KIF20A expression on therapeutic response remains unexplored.

In future studies, we will continue to delve into the molecular mechanisms underlying KIF20A’s role at the cell cycle-immune crosstalk axis and facilitate its clinical translation. Furthermore, future investigations should incorporate a broader spectrum of immune markers, including PD-1 to assess T-cell exhaustion, CD3 and CD4 for broader T-cell context, and markers for myeloid-derived suppressor cells (MDSCs), to more fully delineate the immunomodulatory landscape associated with KIF20A overexpression. By integrating experimental approaches such as organoid models and single-cell sequencing, we will further explore KIF20A’s regulatory roles in shaping the immune microenvironment across spatiotemporal dimensions.

## Conclusion

This study establishes KIF20A as a critical driver of primary anti-PD-1 resistance and poor prognosis in NSCLC. Through integrated bioinformatics and clinical validation, we demonstrate that KIF20A overexpression suppresses PD-L1 expression in tumor cells, shapes an immunosuppressive tumor microenvironment, and independently predicts adverse outcomes in immunotherapy-treated patients. The inverse correlation between KIF20A and PD-L1, coupled with the significantly reduced survival in patients with combined KIF20A-high/PD-L1-low profiles, underscores KIF20A’s dual role as both a prognostic biomarker and a therapeutic target. These findings suggest KIF20A inhibition as a promising strategy to overcome PD-1 blockade resistance and support the development of future combination regimens to enhance immunotherapy efficacy in NSCLC.

## Supplementary Information

Supplementary Material 1.

Supplementary Material 2.

## Data Availability

No datasets were generated or analysed during the current study.

## Abbreviations

ALK: Anaplastic Lymphoma Kinase
CAF: Cancer-Associated Fibroblast
DCR: Disease Control Rate
DDR: DNA Damage Response
DEGs: Differentially Expressed Genes
FP: First Progression
ICI: Immune Checkpoint Inhibitor
IHC: Immunohistochemistry
IRS: Immunoreactive Score
LUAD: Lung Adenocarcinoma
LUSC: Lung Squamous Cell Carcinoma
MDSC: Myeloid-Derived Suppressor Cell
NSCLC: Non-Small Cell Lung Cancer
ORR: Objective Response Rate
PFS: Progression-Free Survival
PPS: Post-Progression Survival
TME: Tumor Microenvironment
Tregs: Regulatory T Cells
