# Supplementary material for: KIF20A as a driver of anti-PD-1 resistance via PD-L1 downregulation in NSCLC: a biomarker validation and tumor microenvironment analysis
Source: BMC Cancer. 2025 Nov 20;25:1794. doi: 10.1186/s12885-025-15221-6 (PMC12636182; doi:10.1186/s12885-025-15221-6)
Supplement: Supplementary file 2 — Supplementary Material 2. [file 12885_2025_15221_MOESM2_ESM.docx]

**Supporting Information**

**KIF20A-Driven Suppression of Tumor PD-L1 Underlies Primary Anti-PD-1 Resistance and Poor Prognosis in NSCLC Patients**

Tianyu Su^b^, Lin Li^a,d^, Chaonan Jing^d^, Lei Chen^c,*^, Xiang Wang^a,d*^

a. Department of Oncology, Xuzhou Central hospital, Xuzhou, Jiangsu 221002, China.

b. Department of Oncology, Shanghai Medical College, Fudan University Shanghai Cancer Center, Fudan University, Shanghai 200032, China.

c. Jiangsu Key Laboratory of New Drug Research and Clinical Pharmacy, Xuzhou Medical University, Xuzhou, Jiangsu, 221004, China

d. The XuZhou Clinical School of Xuzhou Medical University, Xuzhou, Jiangsu 221002, China.

*Corresponding authors.

*Email: wangxiang7726@163.com (X. Wang), chenlei@xzhmu.edu.cn (L. Chen).


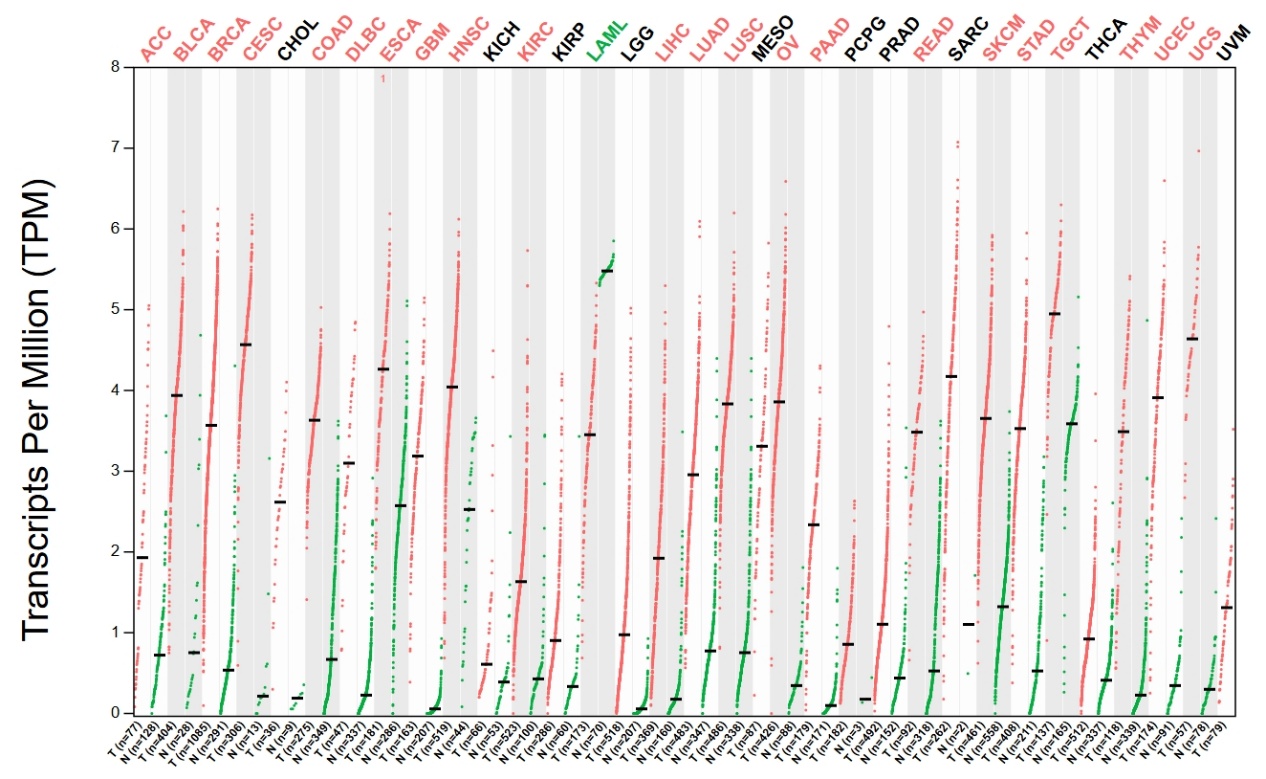


**Figure S1.** Pan-cancer analysis confirms significant upregulation of KIF20A in tumor tissues.


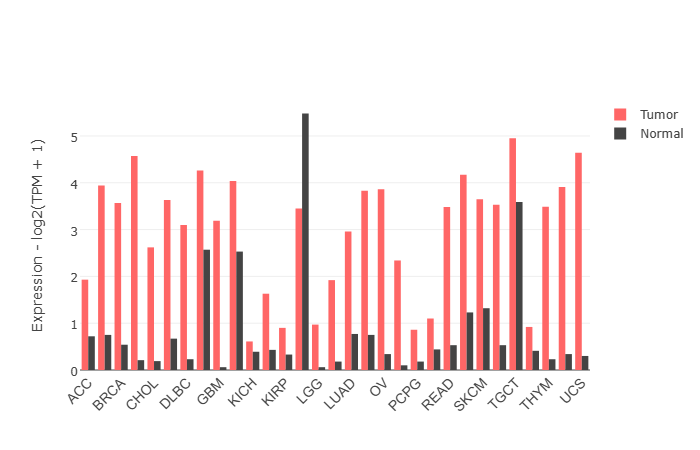


**Figure S2.** Integrated analysis validates elevated KIF20A expression in NSCLC versus normal lung tissues.

**
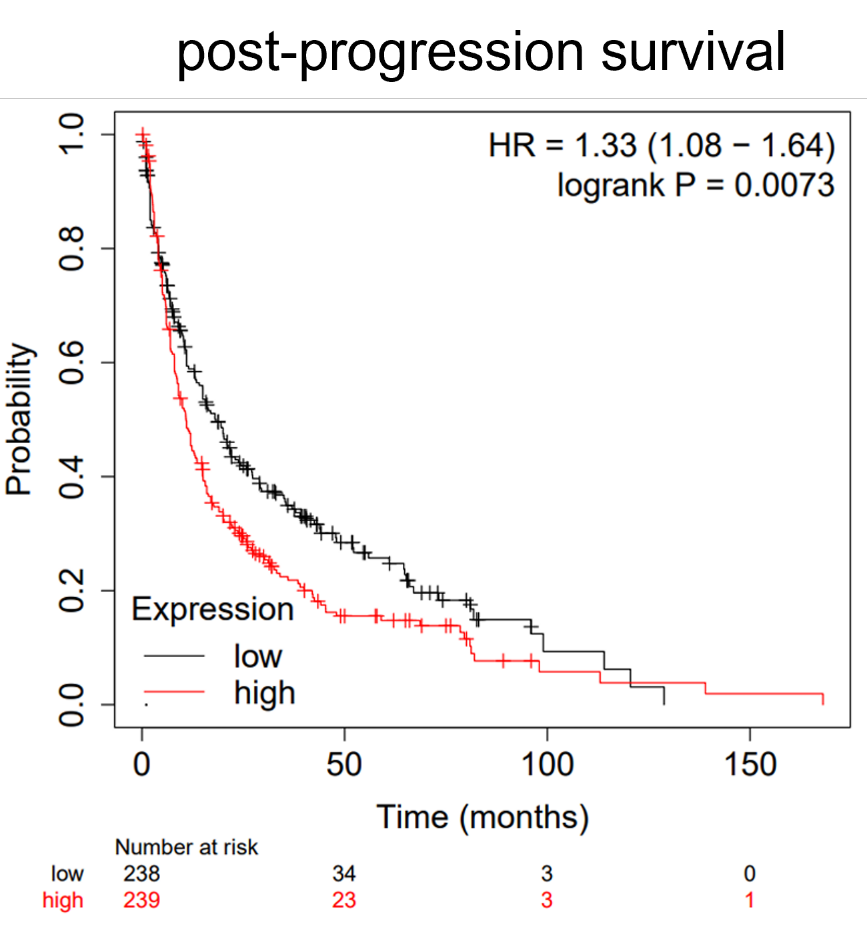
**

**Figure S3.** High KIF20A expression predicts shorter post-progression survival (PPS) in NSCLC.


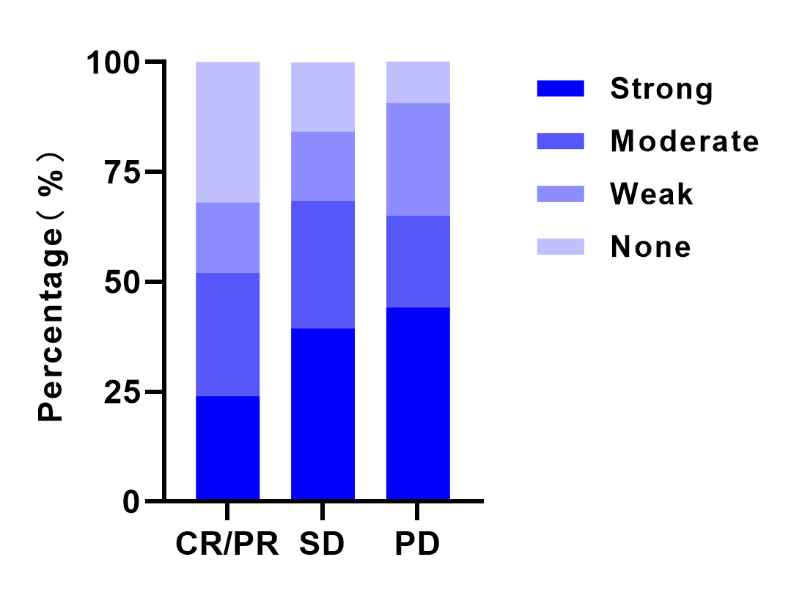


**Figure S4.** Histological association between high expression of KIF20A and primary resistance to anti-PD-1 therapy.
